# Medical and Physical Intelligence

**Published:** 1800-07

**Authors:** 


### MEDICAL AND PHYSICAL INTELLIGENCE.

*Dr. Knebel's Method of Curing the Ague.*—This method is related in his Materials for Medical Science, Vol. I. Part II. and is principally founded on Brunonian principles, or rather on the ſyſtem of medicine, named in German, the Syſtem or Theory of Incitation; (Erregung's Theorie) which, though built upon Brown's ſyſtem, differs from it in ſome points. According to this theory, opium is the chief remedy in intermitting fevers; but Dr. Knebel's method of giving it, is ſo peculiar, that we think proper to mention it. He only adminiſters opium to check the paroxyſm; during the intermiſſion, he gives other excitant medicines, as liquor anodyn. min. Hoffm. eſſence or tincture of valerian, cinamon, &c. with a full diet of meat, wine, &c. In caſes where a kind of ſthenic ſtate is brought on by the uſe of fixed excitantia, bark, &c. opium and other excitants muſt be given with caution, or changed for a few days with gentle ſalts, tartarus tartariſatus, tartar emetic in ſmall doſes, bitters, elixir acid, Haller, &c. Opium, as the univerſal medicine in the paroxyſms, is given in a doſe from two to four grains, which are divided into three parts; the firſt of which is to be taken two hours and a half before the fever comes on, the ſecond about an hour after the firſt, and the laſt not quite half an hour before the paroxyſm commences. The powder of opium is preferable to any other preparation of it; during the paroxyſm, he allows his patients wine and water, or beer; but if the heat is too great, water only. Should opium cauſe too great a ſtupifaction, it may be taken in coffee. When the paroxyſms are repreſſed, it is, however, adviſable to continue the opium for five or ſix times in the ſame manner.

---

Profeſſor Blumenbach has preſented to the Royal Society at Gottingen, ſome phyſiological remarks on a curious animal, which has been lately diſcovered in New South Wales, and brought to England, from whence a ſpecimen was ſent him by Sir Joſeph Banks. This animal, deſcribed already by Dr. Shaw, under the name of *Platypus*, has the ſhape of a ſmall otter, and the beak of a duck. It may certainly be conſidered as an argument in ſupport of the ſcale of nature, if this was an object ſo much deſerving attention as it is thought by ſome. The beak of this animal has alſo a great likeneſs in its internal ſtructure with that of a duck, it being likewiſe provided with a large bundle of nerves from the fifth pair, for the purpoſe of increaſing the ſenſe of feeling ſo neceſſary to them in the ſearch of their nouriſhment. Neverthleſs, it differs in another thing from a bird's beak by having intermaxillar bones, (oſſa

 intermaxillaria)

intermaxillaria) a curious inſtance that Nature follows in the formation of certain animals ſome certain laws, even when this is in other reſpects very anomalous. Dr. Blumenbach calls this animal Ornithorhynchus Paradoxus. *(Goetting Gelehrt Anzeigen,* 1800, No. 62.)

---

Cit. Sedillot the younger, recommends the acetous ether as a gentle anodyne, internally taken; externally applied, it is a powerful diſſolvent without augmenting an inflammation. It produces a gentle warmth on the ſkin, and a ſalutary perſpiration, and is uſed with effect in rheumatic paroxyſm and gout. He obtains this ether by diſtilling equal parts of vinegar and alkohol, and by clearing it from the ſuperfluous acid by the carbonate of potaſs. — *(Hufeland's Neweſt Annals of his French Medicine.)*

---

Profeſſor Tromſdorf at Erfurt, has diſcovered a new earth in a foſſil, commonly called the Saxon beryll, which is found in the mines of the electorate of Saxony. This earth poſſeſſes the following properties. It is of a white colour, and not ſoluble in water; in a freſh ſtate, moiſtened with water, it acquires a degree of expanſibility; and in fire it becomes ſo hard as to ſcratch glaſs. It is diſſolved with eaſe in acids, and compoſes different ſalts with them. Fixed and volatile alkalies have no effect upon it, neither in the humid nor dry way. It has the moſt relation with oxalic acids. The Profeſſor calls this new earth Aguſt-Earth, on account of its having no taſte in all the combinations with acids, by which it materially differs from the glucine. A farther account of it, and likewiſe a deſcription of the foſſil, is to be given in a new Number of the Journal for Pharmacy. *(Fenaer Literture Zeitung.)*

---

Profeſſor Gmelin, at Gottingen, has alſo examined the beryll from Siberia, and nearly found the ſame things as Vauquelin, an account of which will be publiſhed in a ſhort time.

---

The Coccinella Septempunctata has been recommended by a German dentiſt, as a very ſure and certain remedy againſt toothach. It is ſqueezed and rubbed between the thumb and forefinger till the points of the fingers become warm. The gums of the affected tooth, and the tooth itſelf, are now beſmeared with it, on which the pain immediately ceaſes. It was but in few caſes neceſſary to apply it a ſecond time. To preſerve the ſanative power of this inſect, it may be kept in melted wax, ſuet, or in ſugar.

---

The Ruſſian Count Mouſſin Pouſhkin, at Peterſburgh, has diſcovered a new method of forging Platina, totally different from any known before. He is willing to communicate his diſcovery to any artiſt, or ſociety, who will furniſh him with 150lb. of that metal; and he hopes to make it, by his method, an object of trade and manufacture. *Gilbert's Physical Journal,* 1800.

Profeſſor

Professor Hamilton, of Erfurt, attempted to show, in a paper read before the Society of Sciences at Erfurt, on the nature of electric matter, that this consists of light, fire, and phosphoric acid.—*Ibid.*

---

The ingenious Mr. Humboldt has made several interesting experiments, to determine the quantity of carbonic acid, or fixed air, contained in atmospheric air, which was before commonly calculated to be 0,06, and by Mr. Girtanner only 6,01; it was not, however, satisfactorily ascertained. After numerous experiments, Mr. Humboldt found the middle proportion for the temperate zone to be 0,015; the maximum he found was 0,018, at 73° hygrom. Saussure 18° 5′ Reaumur; the minimum 0,05, at 17° Reaumur. Mons. de Saussure observed, even on the summit of Mont Blanc, carbonic acid, which probably had been evolved from some lichens containing carbon, as Lichen sulphureus and rupestris. The quantity of carbonic acid in the air, which Citizen Garnerin brought down with him from a height of 650 toises, was equal to that of the air of Paris. According to these experiments, carbonic acid seems not only to be an adventitious particle of the atmosphere, but to be every where diffused in it; and it is not unlikely we may find hydrogen gas and azote gas combined with it. Carbonic acid could not be traced in rain water. In summer, the atmosphere is found to contain a greater quantity of carbonic acid than at any other time. The proximate causes of its gradual increase and diminution has not yet been discovered. *Ibid.* 1799.

---

The experiments of the same gentleman on the illumination of rotten wood, are as interesting as curious. Carbonic gas, deprived of oxygen gas by means of phosphor, extinguishes the phosphoric light of the wood; some atmospheric air being admitted, it becomes again visible. It does not shine stronger in oxygen gas than in common air; the absorption of it is not considerable, though at last some carbonic acid is perceived. In pure azotic and hydrogen gas, the wood is immediately extinguished; but as soon as atmospheric air is admitted, the lustre is restored. All these airs were purified by phosphor; but to show that the extinction was by no means owing to the evaporated phosphoric acid, he put the wood into atmospheric air impregnated with phosphoric acid, in which it shone with equal strength. The illumination was extinguished by water, or air heated to the degree from 50 tc 32° Reaumur. In cold water, however, it continues to shine a long time. It disappears in alkaline solutions; in alkohol in 6 minutes, and in all acids in 9 or 12 minutes. But we have reason to suppose that Mr. Humboldt did not use for his experiments concentrated sulphuric acid, because this acts upon shining wood with the utmost rapidity, and blackens it, and a hydro-carbon with a great deal of hydrogen is produced. Mr. Humboldt farther observes, that he never could perceive the subterraneous wood in the mines shining, but only that which is exposed to light. But this does not agree with some other observations, obſervations, according to which, wood continually expoſed to light never emits any illumination. *Ibid. Vol. iii. No.* 1.

---

Prince Dimitri Gallitzin has made ſeveral experiments on the germination of ſeeds of garden creſſes, which he ſowed in different gaſes of equal temperature; and the reſults of them are the following: 1. In gas oxygen and azote, he found the ſeeds ſhoot as well as in atmoſpherical air. 2. Gas hydrogen and carbonic acid, both prepared out of different ſubſtances in different ways, ſtopt the germinating of the ſeeds without deſtroying them. They began to ſwell a little, but being during eight days left in the gas, no germ evolved itſelf; meanwhile, the ſeed in the other airs had already emitted four leaves; when atmoſpheric air was admitted, thoſe ſeeds began to produce two leaves in forty-eight hours. 3. Nitrous gas entirely deſtroyed the ſeeds, by blackening and rendering them incapable of germinating in other airs, which is probably owing to the concentrated nitrous acid contained therein. To try whether the ſprouting of potatoes in winter could be prevented by laying them into carbonic gas, he put ſixty potatoes under a bell glaſs filled with carbonic acid, the bottom of which he immerſed in quickſilver, to prevent the admiſſion of atmoſpheric air. The potatoes indeed, though left there for ſix months, did not ſhoot out, but were found in a ſtate quite rotten. *(Vid. Gilbert's Phyſical Journal.* 1800. *4th Vol. No. IV.)*

---

Profeſſor Gmelin, of Göttingen, relates ſeveral experiments with the *tellurium*, which he obtained out of an ore from Tranſylvania, by means of aqua regia. Theſe experiments do not quite agree with thoſe of Klaproth, on account of the metal which Gmelin analyſed being leſs pure. Its ſpecifick weight was only 4,333, and it melted as eaſily as lead; it is very volatile, and evaporates without the leaſt arſenical ſmell, and in a ſtronger heat with a blue flame. Nitric acid diſſolves it with ſome difficulty, but aqua regia with eaſe, and forms a yellow ſolution with it. It is eaſily ſoluble in lixivium, and the ſolution in aqua regia is precipitated with a green colour by lixivium ſanguinis.

---

In explaining the diſappearance of emphyſematous humours, it has been the general opinion, that the abſorbent veſſels had the power of reſorbing and receiving air as ſuch, or in its aëriform ſtate. To prove the truth of this, Dr. Thilow of Erfurt, made the following experiments: He thruſt a trocar into the belly of a healthy dog, and having brought through the opening a quantity of fixed air by means of a ſyringe, he ſhut it immediately with a thick plaſter: the dog's belly became very much inflated, but in one hour and a half the ſwelling was entirely gone and the dog well again. The ſame experiment was repeated with a cat: two hours after ſhe was killed, and in opening her, whole tracts of the abſorbent ſyſtem, in different parts of the body, were found full of air, which was eaſily diſtinguiſhable between the lymph. Dr. Thilow

Thilow was now convinced of the capacity of the abſorbent ſyſtem to receive the air exhaled into the cavities of the body. Perſons, therefore, whoſe digeſtive powers are in perfect and ſound ſtate, are never or very little ſubject to flatulencies, notwithſtanding they have eat victuals containing a great deal of air, which can only be accounted for by the air being received into the abſorbent ſyſtem, mixed with the blood, and exhaled through the ſkin. (*Hufeland's Journal*, *Vol.* ix. *No.* 2.)

---

Dr. Plies relates two inſtances of intermmitting fevers, where flores arnicae, in combination with bark, proved very ſucceſsful, though bark by itſelf, opiates, quaſſia, flores ſalis ammoniaci martiales, were given before without avail. The uſe of arnica in agues has been recommended a long time ago by Collin, Stoll, (Ratio Medendi, Vol. III.) Murray (Apparatus Medicamen, Vol. I. 242, ed Althoff) as a very powerful remedy, but it became of late almoſt out of uſe. Dr. Plies is of opinion, that it may be given with great ſucceſs in ſuch intermittent fevers as have more of the chronic character, where the apyrexies are longer, the froſt more laſting, and the heat leſs; and which are particularly obſervable in weak conſtitutions, and characteriſed by their long duration without the exiſtence of a material cauſe, but merely from an impreſſio nervoſa. (*Ibid.*)

---

Dr. Cappel, of Göttingen, recommends the mineral waters of Driburg in Weſtphalia, which are partly ſaline, partly martial, as the beſt and ſureſt preſervative and remedy againſt ſea ſcurvy. He is going to explain this farther in a pamphlet, which will be publiſhed in a ſhort time.

---

Mr. Lucas, apothecary at Erfurt, has found a ſure and ſimple method of making foul and putrid water again potable, and likewiſe of preſerving water for a long time from being corrupted. This diſcovery, which is particularly intereſting to ſeamen, he propoſes to publiſh for a certain douceur, by way of ſubſcription, for which he may be applied to at Erfurt.

---

The well known Dr. Hahnemann, now reſident at Hamburgh, pretends to have diſcovered a ſpecific remedy, which acts as a very certain preſervative againſt the ſcarlet fever. This diſcovery he is willing to make known to the publick, if he ſhould find three hundred ſubſcribers, of whom each is to pay him a louis-d'or before hand. Upon this every one of them gets a receipt, and along with it a ſmall powder, of which the remedy conſiſts, to try it before its being publiſhed, when an opportunity ſhould offer. This powder is diſſolved in two hundred and forty drops of ſpiritus vini diluted with five parts water; the turbid ſolution which is thus obtained, is a very ſtrong ſolution of the remedy. Of this, one drop is added to one hundred drops of diluted ſpiritus vini, ſhaking it for ſome minutes, which forms the middle ſolution; but this is

ſtill ftill too ftrong to be given. One drop therefore of this, is again dropped into the faid quantity of fpiritus vini diluted, and the mixture fhook for fome minutes. This is the prefervative which is given to thofe who are not yet infected, and will certainly fecure them from the contagion. The dofe for a child of one year old is two drops; for one of two years, three or four drops; from four to fix years, feven or eight drops; for nine years, fourteen to fixteen drops, and then with every year two drops more; however, the dofe muft not exceed forty drops for people from twenty to thirty years old. It is repeated every 72 hours during the time the epidemic is reigning, and continued for fix weeks after it.

---

A very curious inftance of a nearly two years abftinence from all food and drink, is related in two numbers of Hufeland's Practical Journal, Vol. VIII. and IX. No. 2; and a pamphlet has fince been publifhed refpecting this fact, by Dr. Schmidtmann of Melle, in the Bifhoprick of Ofnabruck.

A country girl, fixteen years old, in a village near Ofnabruck, had enjoyed a good ftate of health during her childhood; but at about ten years of age, fhe was feized with epileptic fits, againft which, a number of remedies were employed in vain. Since that time fhe was moftly confined to her bed, particularly in Winter, but in Summer fhe found herfelf a little better. From February 1798, her excretions by ftool and urine began to ceafe, though fhe took now and then a little nourifhment. But from the beginning of April of the fame year, fhe abftained entirely from all food and drink, falling into an uninterrupted flumber almoft fenfelefs, from which fhe only awoke from time to time for a few hours. Her fenfibility was during this time fo great, that the flighteft touch on any part of the body, brought on partial convulfive motions. In this ftate fhe had continued for nearly ten months, when Dr. Schmidtmann faw her firft in March 1799. Though fhe had not taken the leaft nourifhment during all this time, Dr. S. found her, to his utmoft aftonifhment, frefh and blooming. For the laft two months only, the intervals of fleep began to be longer; her fenfes of fight and hearing were in perfect order; but her feeling fhe feemed to have quite loft, as fhe could fuffer pinching of the arms and legs without pain; her gums bled frequently, and the pulfe was fcarcely perceptible in the arms, but beat ftrong and full in the carotids, about 120 in a minute. Dr. S. attempted to make her drink a little milk, but fhe protefted fhe could not fwallow it. The alvine and urinary excretions had quite ceafed.

Although there could hardly be a fufpicion of any kind of impofition, the parents being honeft people; yet, to remove all doubt, fix fworn men were appointed from different places in the neighbourhood to watch her day and night, and inftructions given to them accordingly. This being continued for a fortnight, the men were difmiffed, having given evidence upon oath, that the patient had never taken any food or drink whatever during that time, nor had any excretion by ftool or urine. She had been once very very ill and nearly dying, ſeized with convulſions, feveriſh, and ſometimes in a great ſweat, which had the extraordinary property of turning water black. When Dr. S. ſaw her again, he found her quite recovered, not in the leaſt emaciated, but rather looking luſtier: her gums, however, ſtill frequently bled, and her feeling was not yet returned; but her memory was not impaired, and ſhe amuſed herſelf ſometimes with reading and writing. No alvine and urinary excretion had taken place. Sometimes ſhe was attacked by a ſudden weakneſs, particularly after having bled from the mouth. During laſt ſevere winter, ſhe could not bear the heat of the ſtove, becauſe ſhe felt then faint and oppreſſed,

Dr. Schmidtmann then enters into an enquiry, by what means the patient, in this caſe, was nouriſhed and maintained in that ſtate in which ſhe was found; and having diſcuſſed the matter at large, he is of opinion that ſhe drew, by reſorption, ſuch elementary particles from the atmoſphere, as were ſufficient for the nutrition of the body, and that the excretions were likewiſe replaced by the ſkin.

However incredible and miraculous this fact may ſeem, yet we find ſimilar inſtances recorded by ſeveral authors, viz. by *Haller*, in his *Elementa Phyſiologiae, Tom. VI. Sect. II.* §. 6. *Conf. Memoires de l'Academie de Sciences de Toulouſe, T. I.* 1783; and in *Richter's Library devoted to Surgery*, (in German) *Vol. XII. p.* 184. *Swieten Comment. in Boerhaav. Aph. T. III. p.* 508. *Hiſtoire de l'Acad. Royale de Sciences, l'an* 1769; and in *Hufeland's Art of Prolonging Life, firſt edition, p.* 67. *Halpart. van der Wiel Obſervat. rar. Centur. Poſter.* In the London Magazine for Aug. 1769, there is likewiſe an account of a young woman, twenty-four years of age, who had faſted for two years, and whoſe excretions were alſo entirely ſuppreſſed.

A report has been lately ſpread in one of the newſpapers, that the phyſicians of Oſnabruck have, by an accurate and indefatigable inquiſition, ſucceeded at laſt, in diſcovering the whole fact to be an impoſture of the moſt ſubtle and intricate nature, for the ſake of exciting pity, and getting preſents from people, who came in great numbers from all parts of the neighbourhood to ſee this wonderful girl. It is ſaid, that an account of the whole proceeding will be ſhortly publiſhed, which muſt certainly be very intereſting; and we ſhall not omit to communicate it, after it has been confirmed.

---

Dr. Hildenbrand of Lemberg, in Gallizia, mentions, in Hufeland's Practical Journal, Vol. VII. No. 4. two remedies, which the common people, principally in the eaſtern parts of Gallizia, make general uſe of, to prevent and cure hydrophobia. One conſiſts of a decoction of the wood of Taxus baccata Lin. and the other of a decoction of Lycopodium clavatum Lin. either of which may be uſed externally by waſhing the wound with it, or by giving it internally in large doſes. With the latter, Dr. Hildenbrand himſelf has made ſome trials, as a prophylactic remedy; and it ſeemed to be efficacious, as no perſon who took it, was ſeized with

 the the canine madnefs; but he treated the wounds at the fame time according to Le Roux's method, with butyrum antimonii. It is, however, curious to obferve, that hydrophobia very feldom occurs in this country, though mad wolves frequently come from the neighbouring Carpatic mountains, and attack men and beafts: the common ufe of thofe remedies probably prevents the confequences.

---

Dr. Jördens recommends the following ointment as a very excellent remedy againft chronic ophthalmies of the humid kind. ℞. *Butyr. insuls. unc. jv. mercur. praecip. rubr. dr. ij. intime mistis adde aceti lithargyr. unc. jfs. tere donec omnis humiditas difpareat.* Of this unguent, about as much as the fize of a fmall pea is applied to the internal corner of the eyes every night before going to bed, and the next morning it is wafhed away with cold water: in more obftinate cafes it may be ufed once or twice a day befides. The fpecies of opthalmy, where it is applicable, feems principally to originate from a debility, either partial of the eyes themfelves, or general in the nerves, by which an affluence of acrid humours is produced. The ointment has, therefore, been found ufeful in fuch ophthalmies as arife from too great and continual ufe of the eyes by lucubration, &c. in hereditary ophthalmies, and thofe of children during the time of teething. Venereal opthalmies of this kind were alfo cured by it. *(Arneman's Magazine for Surgery, Vol. II. No. 2.*

---

### VACCINE INOCULATION.

*From the Journal de Paris, of Prarial 23, (June 12, 1800.)*

An account has been given, that on the 18th inftant, thirty children were inoculated (in this city) with the Cow-pox, the matter being fent from England; the procefs according to the method recommended. Symptoms of infection appeared upon nine of the number, with the proper characters, and at the time fpecified by Dr. Pearfon and other members of the London Committee. It is remarkable, that the Englifh phyficians, in their letter to the Committee of Paris, exprefs themfelves happy, confidering the interval between taking the matter and its application, if the inoculation fhould take effect upon one patient out of twenty; — but, on the contrary, nine out of thirty have been properly inoculated.

Upon the 19th, 20th, and 21ft of the fame month, eighteen children, upon whom the firft incifion had been ineffectual, were inoculated afrefh with the matter taken from thofe upon whom it had fucceeded. The Committee continues its experiments, and promifes to give the Public a faithful detail of its proceedings.

Signed, on behalf of the Committee, Thourot.

---

Mr. F. J. Gall, at Vienna, has finifhed a moft elaborate work on the Exercife of the Brain, and on the poffibility of recognifing the feveral Faculties and Propenfities from the Conftruction and Form of the Head and Skull. Mr. Geifweiler, of Parliament Street, has in his poffeffion a part of the manufcript, and feveral drawings, finifhed in the moft correct and elegant ftyle, deferving the attention of the curious. The Author intends to publifh the work at the fame time both in England and Germany.

Dr.

Dr. Beddoes has in the prefs a Collection of Obfervations on the external and internal Ufe of Nitric Acid in Siphylis. He had no intention of publifhing another feparate work on this fubject, but fo many new and interefting facts have been tranfmitted to him, that he very gladly complies with the defire of his correfpondents in fending them to the prefs.

---

Dr. Saunders, phyfician to Guy's Hofpital, will publifh, in a few weeks, a Treatife on the Chemical Hiftory and Medical Powers of fome of the moft celebrated Mineral Waters, together with fome Obfervations on the Cold and Warm Bath, &c.

---

Mr. Davy, Superintendant of the Medical Pneumatic Inftitution, is on the point of publifhing a work, containing a detail of his numerous experiments on Gafes and Refpiration, under the title of *Refearches Chemical and Philofophical chiefly concerning Nitrous Oxide, or Dephlogifticated Nitrous Gas and its Refpiration.*

---

Mr. Stancliffe, of Caius College, Cambridge, F. L. S. and Lecturer of Chemiftry at the Middlefex Hofpital, intends to read a Courfe of Lectures on Chemiftry at the Leverian Mufeum during the enfuing month. The lectures will commence on Wednefday, the fecond of July, at eight o'clock in the evening; and will be continued on every fucceeding Monday and Friday, at the fame hour, till the courfe is finifhed. The fyftem to be purfued in thefe lectures will be that which is adopted by the profeffors in our univerfities, and by modern chemifts; and the plan embraces the hiftory, philofophy, and particular applications of the fcience, both to the arts and agriculture.

---

Dr. Dennison and Dr. Squire will begin, early in July, a Summer Courfe of Lectures on the Theory and Practice of Midwifery, and the Difeafes of Women and Children. Notice of the day will be advertifed in the public papers.

---

On the 17th of May died, at Gottingen, the celebrated Dr. Girtanner, author of many publications in Chemiftry, Medicine, Natural Hiftory, and Politics. His laft work was a reprefentation of Darwin's Syftem of Medicine. He was a native of Swifferland, but had been refident at Gottingen many years.

---

We are forry to have it to ftate to our readers, that on Friday morning, the 27th of June, died (in confequence of an apoplectic fit) the celebrated Mr. Cruikfhank, to whom the world is fo much indebted for many valuable difcoveries in Anatomy and Phyfiology, and who has fo long filled the Teacher's chair in the Theatre of Anatomy, in Great Windmill Street, with fuch refpectability. We are however glad, in having authority to fay, that the Lectures in that fchool will be continued by Mr. Wilson, whofe abilities have been long known, and who, as Colleague with Mr. Cruikfhank, delivered the greateft part of the Lectures laft feafon, with equal credit to himfelf and advantage to the public.

